# Screening fundus photography predicts and reveals risk factors for glaucoma conversion in eyes with large optic disc cupping

**DOI:** 10.1038/s41598-022-26798-4

**Published:** 2023-01-03

**Authors:** Yong Woo Kim, Young In Yun, Hyuk Jin Choi

**Affiliations:** 1 Department of Ophthalmology, Seoul National University College of Medicine, Seoul, Republic of Korea; 2 Department of Ophthalmology, Seoul National University Hospital, Seoul, Republic of Korea; 3 Department of Ophthalmology, Seoul National University Hospital Healthcare System Gangnam Center, 152, Teheran-Ro, Gangnam-Gu, Seoul, 06236 Republic of Korea

**Keywords:** Eye manifestations, Optic nerve diseases

## Abstract

This study aimed to investigate the risk factors for glaucoma conversion and progression in eyes with large optic disc cupping without retinal nerve fiber layer defect (RNFLD). Five hundred forty-two eyes of 271 subjects who had a vertical cup-to-disc ratio (CDR) ≥ 0.6 without RNFLD were enrolled. Characteristics for optic disc configuration (including CDR, vertical cupping, ISNT rule, disc ovality, peripapillary atrophy [PPA]-to-disc area [DA] ratio, and lamina cribrosa pore visibility) and blood vessels (including central retinal vessel trunk [CRVT] nasalization, bayoneting of vessels, baring of circumlinear vessels, history of disc hemorrhage [DH] and vessel narrowing/sclerotic change) were evaluated. From a median follow-up of 11.3 years, 26.6% of eyes (n = 144) developed RNFLD within a median of 5.1 years. Baseline factors, including vertical CDR ≥ 0.7 (hazard ratio [HR] = 2.12), vertical cupping (HR = 1.93), ISNT rule violation (HR = 2.84), disc ovality ≥ 1.2 (HR = 1.61), PPA-to-DA ratio ≥ 0.4 (HR = 1.77), CRVT nasalization ≥ 60% (HR = 1.77), vessel narrowing/sclerotic change (HR = 2.13), DH history (HR = 5.60), and baseline intraocular pressure ≥ 14 mmHg (HR = 1.70) were significantly associated with glaucoma conversion (all *P*s < 0.05). An HR-matched scoring system based on initial fundus photography predicted glaucoma conversion with specificity of 90.4%. Careful examination of the optic nerve head and vascular structures can help to predict the risk of glaucoma conversion in eyes with large optic disc cupping.

## Introduction

Glaucoma is characterized by progressive optic nerve damage that causes progressive visual field (VF) defect, which eventually lead to blindness if left untreated^1^. In most cases, structural changes in the optic disc rim and peripapillary retinal nerve fiber layer (RNFL) precede functional changes in the VF^2–4^. Therefore, early suspicion of structural damage and prediction of ongoing glaucomatous damage provide opportunities to prevent serious visual impairment^5–7^. However, unlike in manifest glaucoma with typical VF defect, assessing glaucoma risk in early suspected cases is clinically challenging. The optic disc has various morphologies among individuals, and the patterns of glaucomatous damage have many variations, making it difficult to make definitive decisions to start treatment^8,9^.

Recently, comprehensive health screening programs including screening fundus photography for early detection and prevention of major diseases are becoming more popular in many countries, and in parallel, the incidental detection of abnormal fundus findings has also been increasing. Large optic disc cupping or large cup-to-disc ratio (CDR) without RNFL defect (RNFLD) is a good example of such findings to which ophthalmologists pay attention. Moreover, ophthalmologists do not infrequently encounter such optic discs in the real practice of fundus examinations, and especially, optic discs with vertical CDR ≥ 0.6 are of special concern because it might reflect a glaucomatous optic disc damage^10^. Although some of these have been described as pseudoglaucomatous physiologic large cups, which are considered to be normal variants, little is known about the long-term natural clinical course and clinical guidelines for programmed follow-up surveillance tests for these eyes have not yet been suggested^11^. Actually, many patients with large CDR without RNFLD have been rountinely followed up under the situation of so-called ‘glaucoma-like disc’ (GLD) or the diagnosis of glaucoma suspect, undergoing wasteful repetitive examinations, including disc photography, RNFL photography, optical coherence tomography (OCT) and automated perimetry for an indefinite period of time. In contrast, RNFLD and/or VF defect develop in some patients with GLD, allowing them to start antiglaucoma medications. Therefore, if we predict the development of glaucoma and know personalized risk factors for glaucoma conversion in eyes with GLD, it is possible to provide each individual with a customized follow-up investigative plan.

Optic disc cupping, estimated by vertical CDR, is widely used by clinicians when assessing glaucoma risk^12^. The Ocular Hypertension Treatment Study (OHTS) demonstrated that a greater vertical CDR is one of the significant predictors of the development of glaucoma^13^. Recent longitudinal studies have also shown that a greater vertical CDR was a risk factor for the incidence of glaucoma or a significant predictor of glaucoma progression judged by VF or disc/peripapillary retina^14^. Therefore, vertical CDR would be a good indicator for predicting glaucoma conversion in eyes with GLD. However, CDR is dependent on the size of the optic disc so that larger optic discs can have physiologically greater vertical CDR^12^. This overlap between normal and glaucoma eyes has diminished the diagnostic capability of vertical CDR, causing us to look for other possible predictors associated with glaucoma development. In line with this situation, screening fundus photography can be easily obtained and provides information not only about the configuration of the optic nerve head (ONH) but also about the hemodynamic status of the retina basd on the morphology of blood vessels or the presence of disc hemorrhage (DH).

In the present study, we assumed that GLD on the way of glaucoma conversion might have personalized additional features (risk factors) in addition to a large vertical CDR on screening fundus photography, suggesting alleged glaucomatous damage or damage reported to be associated with glaucoma development or progression. Accordingly, we aimed to report the long-term natural clinical course of eyes with GLD, investigate personalized risk factors for glaucoma conversion, and suggest a prediction model for glaucoma development in order to guide clinicians to assess the risk for glaucoma conversion in suspected cases and to identify patients who require close monitoring for glaucoma development.

## Methods

The present study was a retrospective, longitudinal, observational study based on the ongoing The Gangnam Eye Cohort Study performed at the Seoul National University Hospital (SNUH) Healthcare System Gangnam Center, which provides comprehensive medical check-ups and screening, after approval by the Institutional Review Board (IRB) of SNUH (IRB No. 1906-141-1043). The study followed the tenets of the Declaration of Helsinki (1964), and written informed consent was waived due to the retrospective design and absence of any additional medical intervention.

### Study subjects and grouping

**Figure 1:**
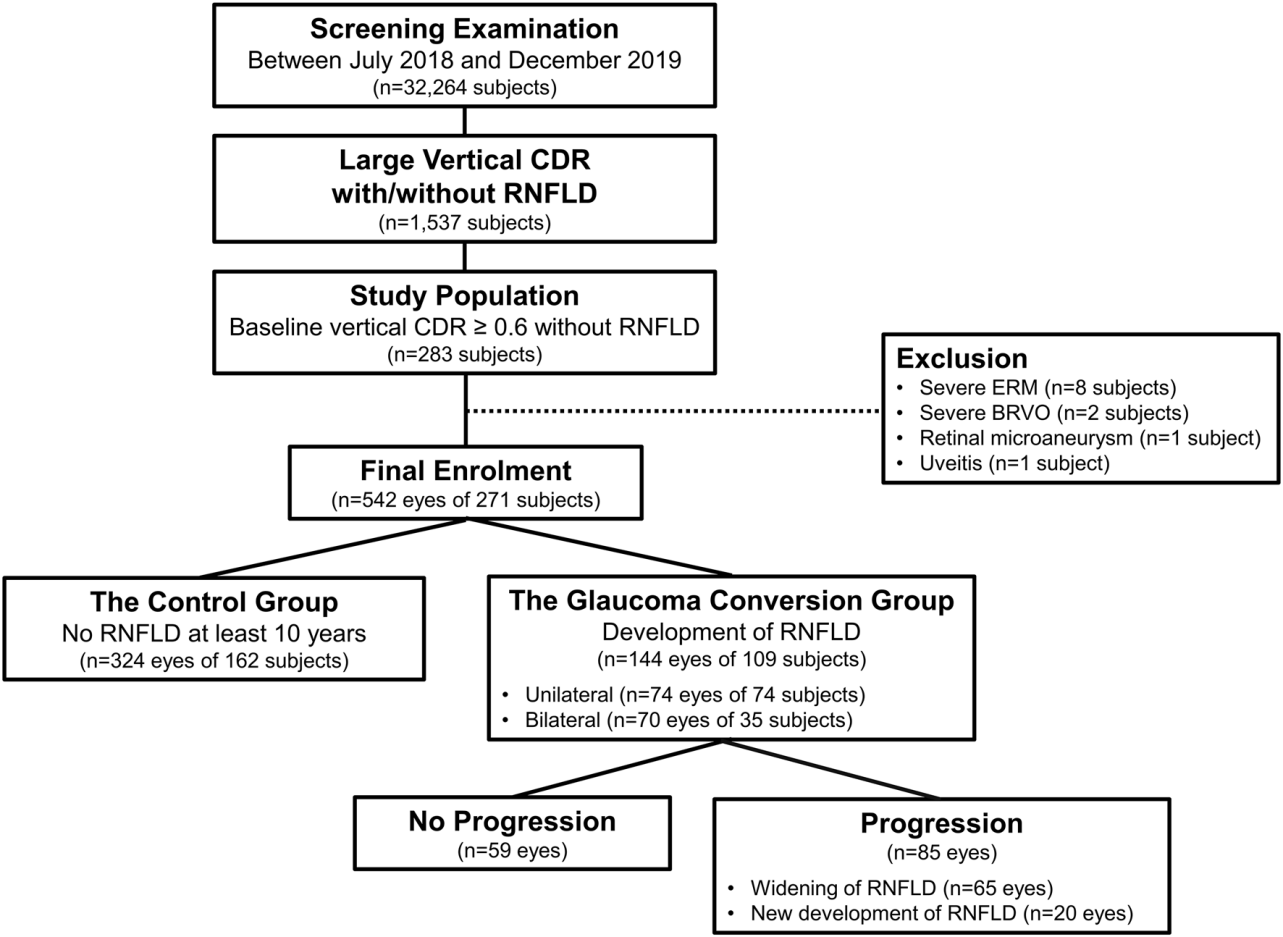
Schematic Diagram of the Subject Enrollment. CDR: cup-to-disc ratio; RNFLD: retinal nerve fiber layer defect; ERM: epiretinal membrane; BRVO: branch retinal vein occlusion.

A schematic diagram for subject enrollment is provided in Fig. 1. Between July 2018 and December 2019, among 32,264 subjects who underwent ophthalmic screening examinations including measurement of intraocular pressure (IOP) by noncontact tonometry (CT-1P; Topcon Inc, Tokyo, Japan) and fundus photography using a 45° digital nonmydriatic fundus camera (TRC-NW8, Topcon Inc.), we consecutively collected 1,537 subjects who had a vertical CDR equal to or greater than 0.6 in at least one eye regardless of RNFLD. Subjects with retinal diseases, including severe epiretinal membrane (*n* = 8), branch retinal vein occlusion (*n* = 2), retinal microaneurysm (*n* = 1), and uveitis (*n* = 1), were excluded.

From thorough review of previous serial fundus images of each subject, we identified subjects who had GLD (vertical CDR ≥ 0.6 without RNFLD) at initial examination and showed a development of RNFLDs during follow-up (the glaucoma conversion group). RNFLDs have been defined as diverging, arched, or wedge shaped and wider than the main retinal vessel at a distance of 1 disc diameter from the edge of the disc^15^. The progression of RNFLD was confirmed when widening of the RNFLD or new development of the RNFLD on the other hemifield was detected. For the control group, we selected subjects who did not show any change in initial GLD status during at least 10 years of follow-up.

The initial fundus images from enrolled subjects were used for further analyses described below (Supplementary Fig. S1). From serial follow-up fundus images, the presence or recurrence of enlargement of the peripapillary atrophy (PPA) area, DH and cotton wool spot (CWS) were monitored.

### Evaluation of optic disc configuration

All the quantitative measurements were performed using built-in calipers in picture archive and communication system (PACS) software. The vertical and horizontal CDRs were defined as the ratio of the cup diameter to the disc diameter in the vertical and horizontal meridians, respectively. Vertical cupping was defined as the vertical CDR being greater than the horizontal CDR. The neuroretinal rim is usually broadest in the inferior region, followed by the superior, nasal, and finally temporal regions (the ISNT rule). Failure to follow this trend in neuroretinal rim configuration was considered a violation of the ISNT rule. If the rim was equally broad in two regions, but the order was not violated, it was considered to be consistent with the ISNT rule. Disc ovality was defined as the ratio between the largest and smallest disc diameters^16^. The area of PPA was plotted using a mouse-driven cursor to trace the PPA and optic disc margin directly on the fundus photograph image. The PPA-to-disc area (DA) ratio was estimated by the ratio of PPA area to DA. The lamina cribrosa (LC) pore was determined to be visible if the eye allowed for clear visualization of the structural details of the LC surface (gray oval pores or fenestrae)^17^.

### Evaluation of vascular structure of optic disc

Central retinal vessel trunk (CRVT) nasalization was estimated as the ratio of the distance between the CRVT and the temporal disc border to the horizontal disc diameter^18^. The bayoneting of blood vessels was defined as the vessels being sharply bent or kinked while passing the edge of the cup^19^. The baring of circumlinear vessels was defined as the arteriole or vein falling to the bottom of the cup or molded onto the sidewall of the enlarging cup, rather than curving to follow the contour of the cup^20,21^. A single vessel that arises directly from the central bifurcation and ascends the temporal rim was excluded from this definition. DHs located on the optic disc tissue (laminar or prelaminar tissue), optic disc margin, or peripapillary retina were counted.

### Evaluation of other possible contributing factors

The fovea-disc angle was defined as the angle between the horizontal line and the axis connecting the fovea to the center of the optic disc on the acquired fundus image. Retinal arterial narrowing/sclerotic changes were determined by a modified Scheie’s classification system to infer the ischemic status of the retina^22^. Diffuse arteriolar narrowing, focal constriction, arteriovenous compression, or copper-wire appearance were considered positive signs. The presence of CWS was also considered an ischemic marker.

The baseline IOP was defined as the IOP measured at the time of enrollment in the study. The mean IOP was defined as the average of the IOP measurements during the study period. The peak IOP was defined as the maximum IOP measurement value during the study period. The IOP fluctuation was defined as the standard deviation of the IOP measurements during the study period. In addition, all of the subjects answered a questionnaire for the presence of systemic diseases, including diabetes mellitus (DM) and systemic hypertension (HTN).

### Data analysis

Two independent ophthalmologists (Y.W.K and Y.I.Y) evaluated the fundus photography and rated the optic disc configuration and vascular structures. If the readings did not match each other, they were determined by consensus between the two ophthalmologists or a third adjudicator (H.J.C). The interrater agreement was obtained by estimation of Cohen’s kappa (for categorical variables) or the intraclass coefficient (ICC, for continuous variables). The vertical CDR showed only moderate interobserver agreement (ICC = 0.635 and 95% CI = 0.414–0.786), but the kappa statistics for vertical CDR $\ge$ 0.7 were almost perfect (Cohen’s kappa = 0.844). Other parameters also showed reliable interobserver agreement (Supplementary Table S1).

Data normality was tested by the Shapiro–Wilk test. Continuous variables for comparison between the groups were analyzed by Student’s t-test for normally distributed data and the Mann–Whitney test for nonnormally distributed data. Categorical variables were compared using the chi-square test. The cutoff values for baseline age (44 years old), CDR (0.7), disc ovality (1.2), PPA-to-DA ratio (0.40), CRVT nasalization (60%), and IOP parameters (baseline IOP [14 mmHg], mean IOP [13 mmHg], peak IOP [16 mmHg], and IOP fluctuation [2.0 mmHg]) of the eyes were identified using a maximal chi-squared method for each variable using the ‘maxstat’ package in R software (R version 3.6.2., available at: http://www.r-project.org; accessed April 2021)^23^.

Survival analysis was performed using the “survival” package in R software. The Kaplan–Meier method was used to estimate the median time to the development of RNFLD and its progression. A Cox proportional hazards (PH) model was used to investigate the clinical factors (age, sex, vertical CDR, vertical cupping, ISNT rule violation, disc ovality, PPA-to-DA ratio, PPA enlargement, LC pore visibility, CRVT nasalization, bayoneting of blood vessels, baring of circumlinear vessels, history of DH and CWS, fovea-disc angle, vessel narrowing/sclerotic change, baseline IOP, mean IOP, peak IOP, IOP fluctuation, DM and HTN) associated with glaucoma conversion in the first and fellow eyes and progression of RNFLD. The Cox PH model was clustered by the subjects, as the study eyes included both eyes in some subjects. The stepwise function was used to fit the best multivariable model with the lowest Akaike information criterion (AIC) values. The performance of the baseline scoring system for the prediction of the risk of glaucoma conversion was determined by calculating the areas under receiver operating characteristic curves (AUROCs). A receiver operating characteristic (ROC) analysis was performed using the ‘‘pROC’’ package in R software (R version 3.6.2., available at: http://www.r-project.org; accessed April 2021)^24^. The best cutoff value was selected according to the Youden index value (which maximizes the value of ‘sensitivity + specificity-1’)^25^.

The false discovery rate was controlled for using the Holm-Bonferroni method. Except where stated otherwise, the data are presented as the mean ± standard deviation, and the level of statistical significance was set at *P* < 0.05. All the statistical analyses were performed with R software (R version 3.6.2., available at: http://www.r-project.org; accessed April 2021).

## Results

The present study finally enrolled 542 eyes of 271 subjects, and a total of 5,126 fundus photograph images were evaluated during a median longitudinal follow-up of 11.3 years. Among these eyes, 144 eyes of 109 subjects (26.6%) developed RNFLDs (the glaucoma conversion group). The first RNFLDs were developed unilaterally in 100 subjects and in both eyes simultaneously in 9 subjects. The median time to the development of the first RNFLDs was 5.1 years (range: 0.7–13.9 years). During the follow-up, 26 of 100 subjects with unilateral RNFLDs showed additional RNFLDs in the fellow eyes. Finally, 74 eyes of 74 subjects showed unilateral glaucoma conversion, and 70 eyes of 35 subjects showed bilateral glaucoma conversion at the last follow-up. The remaining 324 eyes of 162 subjects were free of RNFLDs during a median follow-up period of 12.2 years and were set as the control group (Fig. 1).

### Subject demographics

The baseline characteristics of the participants are provided in Table 1. At baseline, the eyes of the glaucoma conversion group had significantly greater vertical CDR (medians, 0.64 vs. 0.62, *P* < 0.001), proportions of vertical CDR ≥ 0.7 (22.9% vs. 3.7%, *P* < 0.001), proportions of vertical cupping (52.8% vs. 32.4%, *P* < 0.001), and proportions of PPA-to-DA ratio ≥ 0.40 (25.7% vs. 11.4%, *P* < 0.001) than control eyes. Moreover, retinal arterial narrowing/sclerotic signs (43.1% vs. 12.3%, *P* < 0.001) was more frequently observed on the initial fundus images from the glaucoma conversion group. During the follow-up period, the eyes of the glaucoma conversion group also showed a significant PPA enlargement rate (20.8% vs. 1.2%, *P* < 0.001) and a more frequent history of DH (34.7% vs. 1.2%, *P* < 0.001).

**Table 1:** Subject demographics and baseline findings.

	The Glaucoma conversion group (*n* = 144 eyes of 109 subjects)	The Control group (*n* = 324 eyes of 162 subjects)	*P*-values
Age, *yrs*	48.3 (42.2–53.9)	46.7 (40.8–52.6)	0.16*
Gender, female, *n (%)*	38 (34.9)	59 (36.4)	0.79^†^
FU period, *yrs*	11.6 (9.9–13.7)	12.4 (11.0–13.7)	0.07*
**Vertical CDR**	**0.64 (0.62–0.69)**	**0.62 (0.59–0.65)**	< 0.001*
**Vertical CDR < 0.7, *n (%)***	**111 (77.1)**	**312 (96.3)**	** < 0.001**^†^
**Vertical CDR** $\ge$ **0.7, *n(%)***	**33 (22.9)**	**12 (3.7)**	
**Vertical cupping, *n (%)***	**76 (52.8)**	**105 (32.4)**	** < 0.001**
ISNT rule violation, *n (%)*	11 (7.6)	5 (1.5)	0.002
Disc ovality	1.14 (1.07–1.19)	1.11 (1.06–1.17)	0.01*
Disc ovality < 1.2, *n (%)*	109 (75.7)	267 (82.4)	0.12^†^
Disc ovality $\ge$ 1.2, *n (%)*	35 (24.3)	57 (17.6)	
PPA-to-DA ratio	0.21 (0–0.40)	0.16 (0–0.28)	0.004*
**PPA-to-DA ratio < 0.40, *n (%)***	**107 (74.3)**	**287 (88.6)**	** < 0.001**^**†**^
**PPA-to-DA ratio** $\ge$ **0.40, *n (%)***	**37 (25.7)**	**37 (11.4)**	
**PPA enlargement**	**30 (20.8)**	**4 (1.2)**	** < 0.001**
LC pore visibility, *n (%)*	38 (26.4)	71 (21.9)	0.35^†^
CRVT nasalization, *%*	62.1 (54.0–71.4)	59.3 (52.2–68.6)	0.01*
CRVT nasalization < 60%, *n (%)*	64 (44.4)	176 (54.3)	0.06^†^
CRVT nasalization $\ge$ 60%, *n (%)*	80 (55.6)	148 (45.7)	
Bayoneting of blood vessels, *n (%)*	93 (64.6)	248 (76.5)	0.01^†^
Baring of circumlinear vessels, *n (%)*	26 (18.1)	58 (17.9)	> 0.99^†^
**DH history, *n (%)***	**50 (34.7)**	**4 (1.2)**	** < 0.001**^**†**^
Foveo-disc angle	6.71 (4.93–9.34)	6.96 (4.71–9.36)	0.29*
**Vessel narrowing/sclerotic change, *n* (*%*)**	**62 (43.1)**	**40 (12.3)**	** < 0.001**^**†**^
Cotton wool spot history, *n (%)*	5 (3.5)	4 (1.2)	0.14^†^
Baseline IOP, mmHg	15 (13–16)	14 (12–16)	0.03*
Baseline IOP < 14 mmHg, *n (%)*	45 (31.2)	144 (44.4)	0.01
Baseline IOP $\ge$ 14 mmHg, *n (%)*	99 (68.8)	180 (55.6)	
Mean IOP, mmHg	14.0 (13.0–16.0)	13.9 (12.2–15.4)	0.04^‡^
**Mean IOP < 13 mmHg, *n (%)***	**29 (20.1)**	**117 (36.1)**	**0.001**
**Mean IOP** $\ge$ **13 mmHg, *n (%)***	**115 (79.9)**	**207 (63.9)**	
**Peak IOP, mmHg**	**17 (15–19)**	**16 (14–18)**	< 0.001*
Peak IOP < 16 mmHg, *n (%)*	49 (34.0)	156 (48.1)	0.006
Peak IOP $\ge$ 16 mmHg, *n (%)*	95 (66.0)	168 (51.9)	
IOP fluctuation, mmHg	1.7 (1.0–2.0)	1.6 (1.2–2.0)	0.40*
**IOP fluctuation < 2.0 mmHg, *n (%)***	**84 (58.3)**	**250 (77.2)**	** < 0.001**
**IOP fluctuation** $\ge$ **2.0 mmHg, *n (%)***	**60 (41.7)**	**74 (22.8)**	
DM, *n (%)*	13 (11.9)	11 (6.8)	0.14^†^
HTN, *n (%)*	32 (29.4)	36 (22.2)	0.18^†^

Values are median (interquartile range). *Comparison was performed using Mann–Whitney test. ^†^Comparison was performed using chi-square test. ^**‡**^Comparison was performed using student t-test. Statistically significant *P*-values after Holm-Bonferroni method are shown in bold. RNFL: retinal nerve fiber layer; FU: follow-up; HTN: hypertension; DM: diabetes mellitus; IOP: intraocular pressure; CDR: cup-to-disc ratio; ISNT: inferior-supeior-nasal-temporal; PPA: peripapillary atrophy; DA: disc area; LC: lamina cribrosa; CRVT: central retinal vessel trunk; DH: disc hemorrhage.

The eyes in the glaucoma conversion group, compared to those in the control group, had significantly greater peak IOP (17 mmHg vs. 16 mmHg, *P* < 0.001) and proportions of IOP parameters greater than the cutoff points: mean IOP $\ge$ 13 mmHg (79.9% vs. 63.9% *P* = 0.001) and IOP fluctuation $\ge$ 2.0 mmHg (41.7% vs. 22.8%, *P* < 0.001).

There were no significant differences in the follow-up period, age, sex, or history of DM or HTN between the two groups.

### Cox PH model for the risk of glaucoma conversion

The multivariable model showed that vertical CDR $\ge$ 0.7 (hazard ratio [HR] = 2.12, *P* = 0.004), vertical cupping (HR = 1.93, *P* < 0.001), violation of the ISNT rule (HR = 2.84, *P* = 0.002), disc ovality $\ge$ 1.2 (HR = 1.61, *P* = 0.04), PPA-to-DA ratio $\ge$ 0.4 (HR = 1.77, *P* = 0.005), nasalization of CRVT $\ge$ 60% (HR = 1.77, *P* = 0.009), history of DH (HR = 5.60, *P* < 0.001), retinal arterial narrowing/sclerotic changes (HR = 2.13, *P* < 0.001) and baseline IOP $\ge$ 14 mmHg (HR = 1.70, *P* = 0.01) were significantly associated with the risk of glaucoma conversion (Table 2).

**Table 2:** Cox proportional hazard model for the risk of glaucoma conversion.

Variable	HR (95% CI)	*P*-value
Age $\ge$ 44	1.38 (0.88–2.17)	0.16
Gender, female	1.53 (0.97–2.40)	0.07
**Vertical CDR** $\ge$ **0.7**	**2.12 (1.27–3.54)**	**0.004**
**Vertical cupping**	**1.93 (1.33–2.78)**	** < 0.001**
**ISNT rule violation**	**2.84 (1.46–5.55)**	**0.002**
**Disc ovality** $\ge$ **1.2**	**1.61 (1.03–2.51)**	**0.04**
**PPA-to-DA ratio** $\ge$ **0.4**	**1.77 (1.19–2.65)**	**0.005**
LC pore visibility	0.68 (0.43–1.08)	0.10
**CRVT nasalization** $\ge$ **60%**	**1.77 (1.15–2.71)**	**0.009**
**DH history**	**5.60 (3.45–9.11)**	** < 0.001**
**Vessel narrowing/sclerotic change**	**2.13 (1.40–3.23)**	** < 0.001**
Bayoneting of blood vessels	0.76 (0.50–1.16)	0.20
**Baseline IOP **$\ge$** 14 mmHg**	**1.70 (1.11–2.61)**	**0.01**
IOP fluctuation $\ge$ 2 mmHg	1.42 (0.97–2.09)	0.07

RNFL: retinal nerve fiber layer; HR: hazard ratio; CI: confidence interval; FU: follow-up; IOP: intraocular pressure; CDR: cup-to-disc ratio; ISNT: inferior-supeior-nasal-temporal; PPA: peripapillary atrophy; DA: disc area; LC: lamina cribrosa; CRVT: central retinal vessel trunk; DH: disc hemorrhage.

Significant values are in bold.

### Cox PH model for the risk of RNFL defect on the fellow eyes

In 26 subjects (23.9%) in the glaucoma conversion group, new RNFLDs developed in the fellow eyes within 3.1 years (range: 1.3–9.3 years) of median follow-up after the development of the first RNFLD in one eye. The multivariable model revealed that the risk of RNFLD in the fellow eye significantly increased when the fellow eye had baseline factors such as a history of DH (HR = 2.64, *P* = 0.02) during follow-up and retinal arterial narrowing/sclerotic signs (HR = 4.64, *P* = 0.002) (Supplementary Table S2).

### Cox PH model for the risk of the progression of RNFL defect

Of the 144 eyes with RNFLDs, 85 eyes (59.0%) showed progression within 3.1 years of median follow-up (range: 0.7–10.9 years) after the first detection of RNFLDs: 65 eyes (76.5%) showed widening of the RNFLD, and a new RNFLD developed in the other hemifield in the remaining 20 eyes (23.5%). The multivariable model demonstrated that an absence of bayoneting of blood vessels at baseline (HR = 0.51, *P* = 0.009), history of DH (HR = 2.32, *P* < 0.001) and absence of CWS history (HR = 8.4E–09, *P* =  < 0.001) significantly increased the risk of RNFLD progression (Supplementary Table S3).

### Scoring system to predict the risk of glaucoma conversion

To develop a scoring system for assessing the risk of glaucoma conversion, baseline factors were included in the multivariable Cox PH model (Supplementary Table S4). Based on this model, an HR-matched weight was assigned to each variable that was significant, and the scoring system was constructed as follows:

$$\begin{gathered} Risk\;score = 1.59 \times \left( {age \ge 44\;years} \right) + 3.10 \times \left( {vertical\;CDR \ge 0.7} \right) + 1.62 \times \left( {vertical\;cupping} \right) \hfill \\ \quad \quad \quad \quad \quad + \; 2.64 \times \left( {the\;violation\;of\;the\;ISNT\;rule} \right) + 1.66 \times \left( {disc \; ovality \ge 1.2} \right) + 1.63 \times \left( {{PPA}\text{-}to{\text{-}}{DA}\;ratio \ge 0.4} \right) \hfill \\ \quad \quad \quad \quad \quad + \; 2.23 \times \left( {CRVT\;nasalization \ge 60\% } \right) + 3.12 \times \left( {retinal\;arterial\;narrwing\;or\;sclerotic\;change} \right) \hfill \\ \quad \quad \quad \quad \quad - 0.45 \times (bayoneting\;of\;blood\;vessels) + 1.60 \times \left( {IOP \ge 14\;{\text{mmHg}}} \right) \hfill \\ \end{gathered}$$

**Figure 2:**
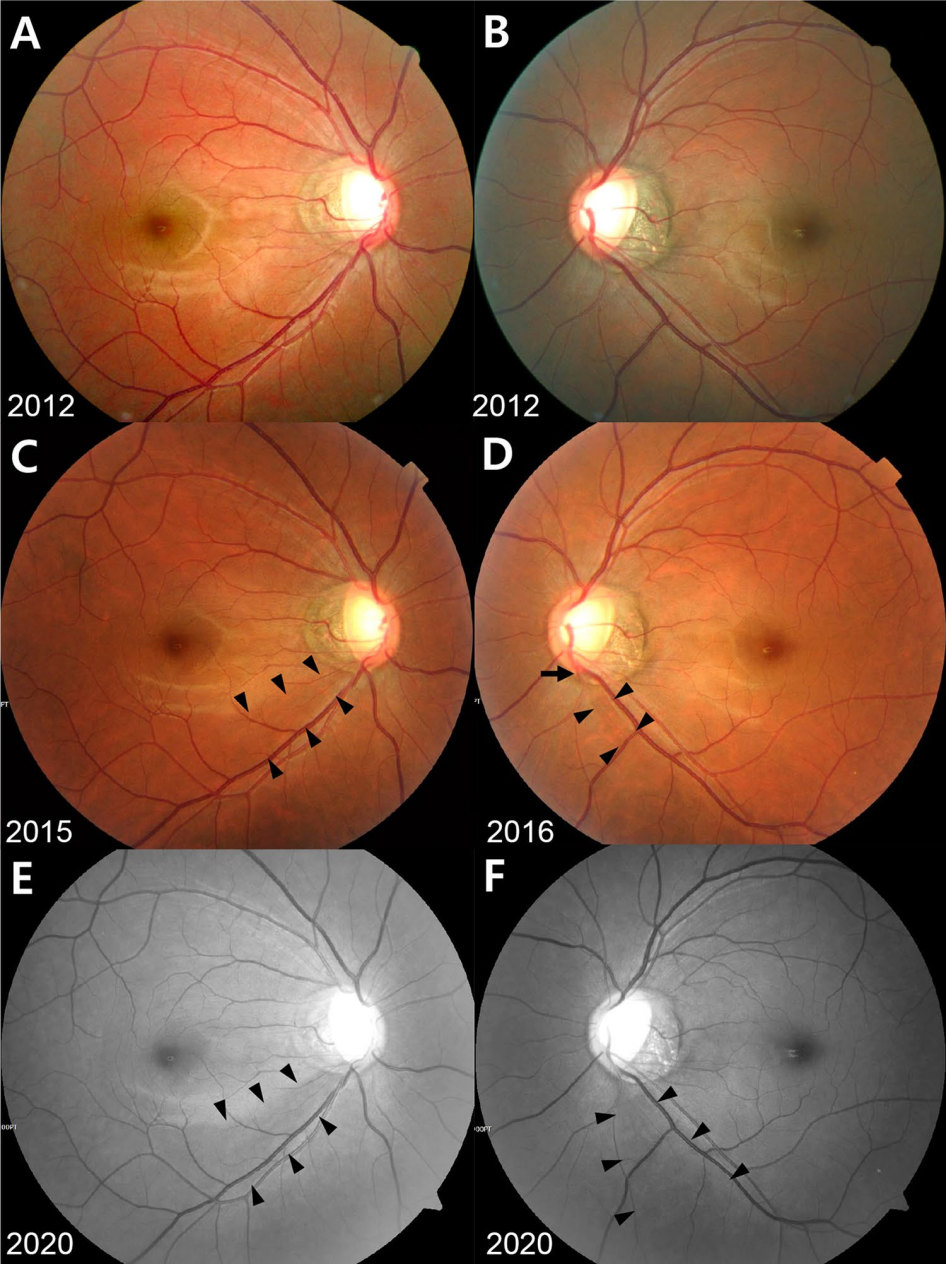
Representative Case No. 1 in the Glaucoma Conversion Group. (**A**, **C**, **E**) right eye, (**B**, **D**, **F**) left eye. Baseline fundus photographs of 32-year-old man taken at a health screening center showed the large optic disc cupping with vertical cupping and the bayoneting of blood vessels but the absence of retinal nerve fiber layer defect (RNFLD) in both eyes. The vertical cup-to-disc ratio, the disc ovality, the peripapillary atrophy area-to-disc area ratio, the central retinal vessel trunk nasalization and the baseline intraocular pressure (IOP) were 0.71, 1.60, 0.97, 73% and 18 mmHg in the right eye and 0.73, 1.45, 0.72, 69% and 17 mmHg in the left eye, respectively. The baseline risk score was 11.39 in both eyes (**A** and **B**: 2012). In 2014, developments of inferotemporal RNFLDs were suspected in both eyes, which became more evident in the right eye (arrowheads) in the following year (**C**: 2015). Thereafter, the patient has been regularly followed up at a glaucoma clinic at a tertiary referral hospital. In 2016, the RNFLD in the left eye became more evident (arrowheads) with the development of the optic disc hemorrhage (arrow) (**D**). During the follow up period at the health screening center, the mean IOP, the peak IOP and the IOP fluctuation were 18.3, 20 and 1.03 mmHg in the right eye and 17.0, 19 and 1.41 mmHg in the left eye, respectively. The most recent RNFL photographs taken at the glaucoma clinic showed definite deepening as well as widening of the RNFLDs in both eyes (**E** and **F**: 2020). However, anti-glaucoma medication has not been started yet as automated perimetry has not revealed typical glaucomatous visual field defects in both eyes.

**Figure 3:**
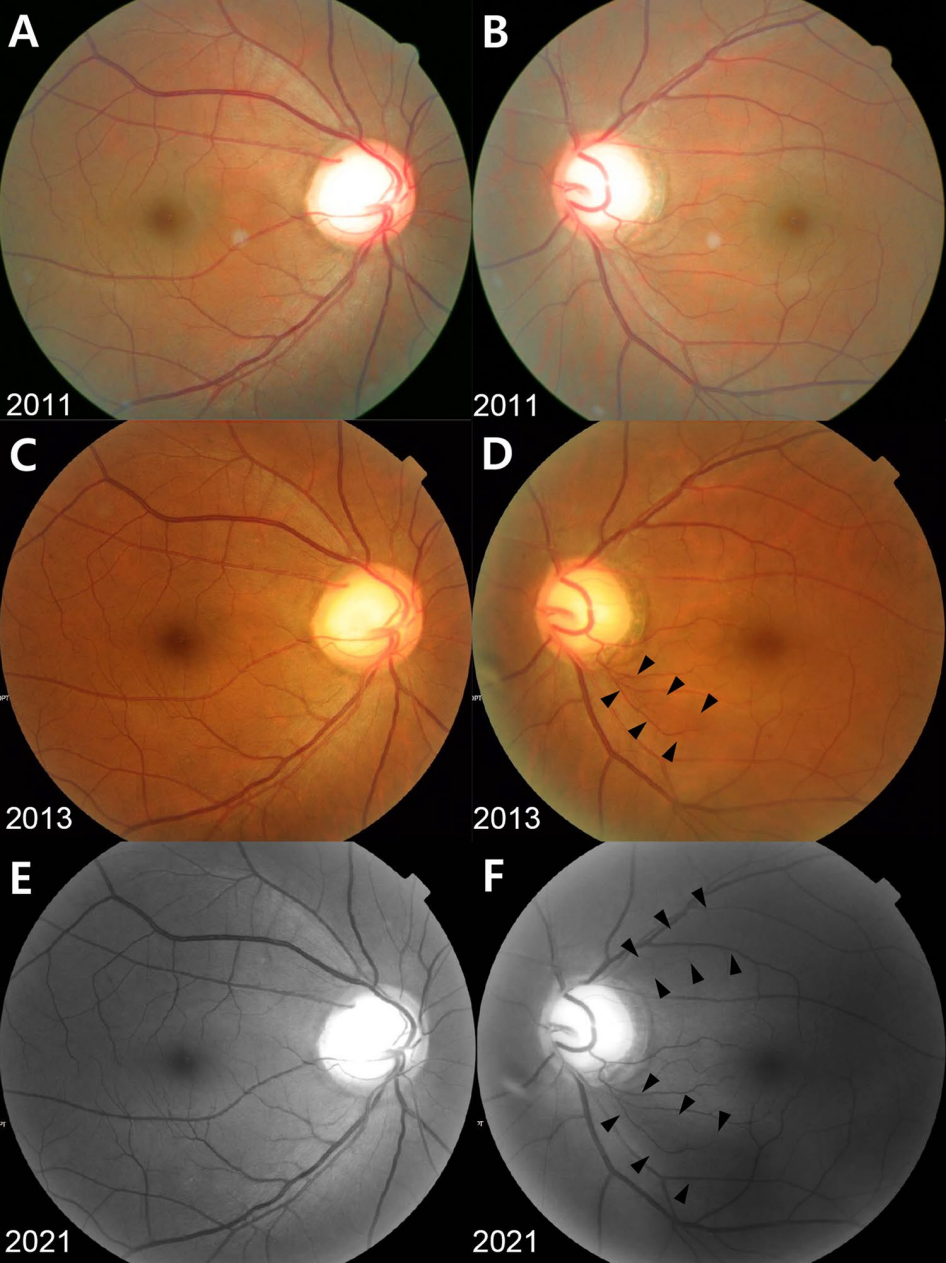
Representative Case No. 2 in the Glaucoma Conversion Group. (**A**, **C**, **E**) right eye, (**B**, **D**, **F**) left eye. A 45-year-old man visited a health screening center in 2011. Screening fundus photographs showed the large optic disc cupping with violation of the ISNT rule in both eyes. The right eye showed horizontal cupping without the peripapillary atrophy (PPA), whereas the left eye showed vertical cupping with small area of PPA (PPA area-to-disc area ratio = 0.24) and the bayoneting of a superior optic disc vessel. The vertical cup-to-disc ratio, the disc ovality, the central retinal vessel trunk nasalization and the baseline intraocular pressure (IOP) were 0.80, 1.06, 70% and 17 mmHg in the right eye and 0.83, 1.15, 63% and 18 mmHg in the left eye, respectively. There was mild sclerosis of the retinal arteries in both eyes. The baseline risk score was 14.28 in the right eye and 15.45 in the left eye, respectively (**A** and **B**). In the following year, the thinning of inferotemporal retinal nerve fiber layer was suspected only in the left eye, which became more evident in 2013 (**C** and **D**). During the follow up period, the left eye showed very slow progression of inferotemporal retinal nerve fiber layer defect (RNFLD) and an additional RNFLD in the superotemporal area. The mean IOP, the peak IOP and the IOP fluctuation were 15.7, 18 and 1.97 mmHg in the right eye and 16.5, 21 and 2.74 mmHg in the left eye, respectively. In 2020, the patient was eventually referred to a glaucoma clinic at a tertiary referral hospital and anti-glaucoma medication has been administered in the left eye since a glaucoma specialist confirmed definite thinning of superotemporal and inferotemporal RNFL on the optical coherence tomography and corresponding visual field defect on the automated perimetry. The most recent RNFL photographs taken at the glaucoma clinic in 2021 showed large superotemporal and inferotemporal RNFLDs in the left eye, while there was no definite RNFLD in the right eye (**E** and **F**).

**Figure 4:**
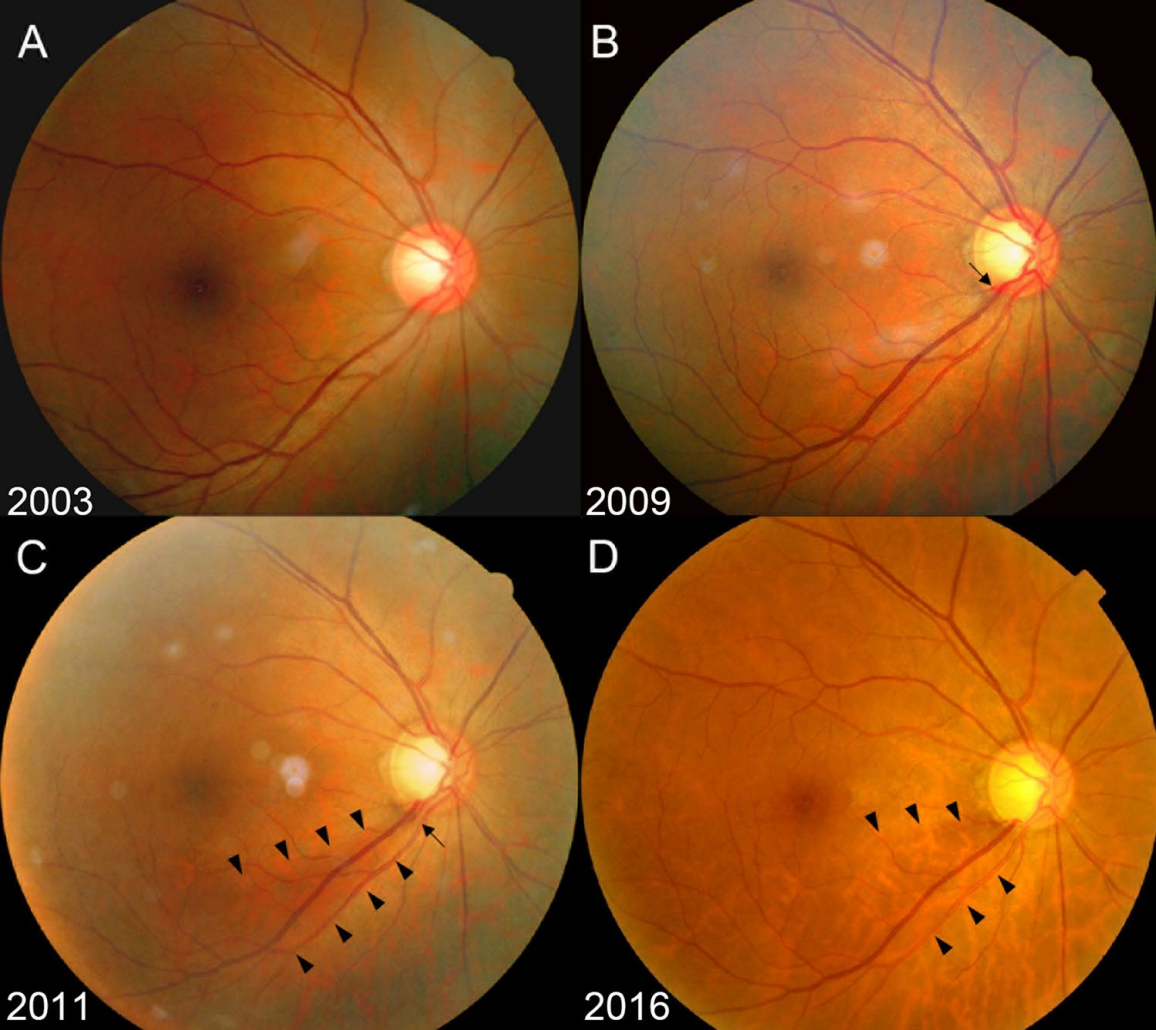
Representative Case No. 3 in the Glaucoma Conversion Group. Serial fundus photographs (**A**: 2003; **B**: 2009; **C**: 2011; **D**: 2016) of the right eye of 53-year-old woman with baseline intraocular pressure (IOP) of 14 mmHg. Note the large optic disc cupping (vertical cup-to-disc ratio [CDR] = 0.61) with vertical cupping and violation of the ISNT rule without retinal nerve fiber layer defect (RNFLD) at baseline. The disc ovality, peripapillary atrophy area-to-disc area ratio, and central retinal vessel trunk nasalization were 1.11, 0.06, and 73%, respectively. The baseline risk score was 9.68 (**A**). Initial disc hemorrhage (DH, arrow) developed at the inferotemporal neuroretinal rim and increased CDR with the thinning of inferotemporal RNFL was suspected **(B)**. The definite RNFLD (outlined by arrowheads) was first detected at the inferotemporal region 2 years later with the concurrent DH (arrow) (**C**). The RNFL showed a progressive thinning afterward, with definite loss of the inferotemporal neuroretinal rim (**D**). During the follow up period from November 2003 to January 2016, the mean IOP was 13.5 mmHg with the IOP fluctuation of 1.3 mmHg and peak IOP of 15 mmHg.

**Figure 5:**
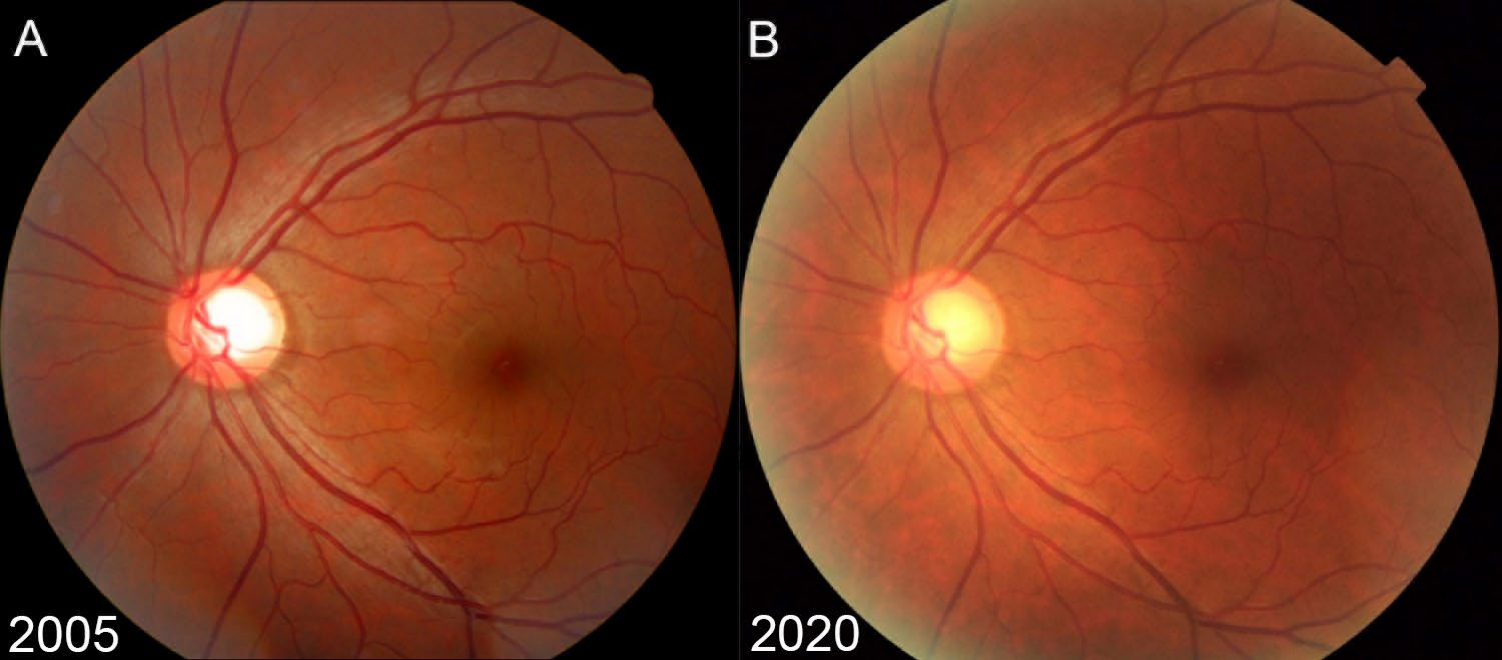
Representative Case in the Control Group. Serial fundus photographs (**A**: 2005; **B**: 2020) of the left eye of a 41-year-old man with baseline intraocular pressure (IOP) of 13 mmHg. Although the baseline vertical cup-to-disc ratio was 0.64, the eye did not show vertical cupping, violation of the ISNT rule, peripapillary atrophy or tilted disc configuration. There was no vessel narrowing or sclerotic changes, and the nasalization of central retinal vessel trunk was 49%. The baseline risk score was 0 **(A)**. The subject did not show any retinal nerve fiber layer defects during the 15 years of follow-up **(B)**.

Each variable has a value of 1 if the condition is met and 0 otherwise. The AUROC of the risk score for glaucoma conversion was 0.793 (Supplementary Fig. S2). The best cutoff score was 7.3, with sensitivity of 43.1% and specificity of 90.4%. Representative cases of glaucoma conversion and the control are provided in Figs. 2, 3, 4, 5.

## Discussion

Through long-term surveillance using screening fundus photography, we found that glaucoma can develop from GLD (glaucoma conversion) at a median follow-up time of 5.1 years (range: 0.7–13.9 years). Vertical CDR $\ge$ 0.7, vertical cupping, violation of the ISNT rule, disc ovality $\ge$ 1.2, PPA-to-DA ratio $\ge$ 0.4, nasalization of CRVT $\ge$ 60%, history of DH, retinal arterial narrowing/sclerotic changes and baseline IOP $\ge$ 14 mmHg were significant risk factors for glaucoma conversion by the multivariable model. Moreover, we devised a meaningful scoring system in which HR-matched variables from the initial fundus photography were used to predict glaucoma conversion.

Clinical examination of the ONH is the principal procedure in the management of glaucoma. It can be easily performed with high accessibility and cost-effectiveness for glaucoma screening. Optic disc cupping, often estimated by the CDRs, is the most common sign for general ophthalmologists to consider glaucomatous ONH changes. However, due to the high interindividual variability of the ONH configuration and its dependence on the size of the optic disc, CDRs have limited value for the screening and diagnosis of glaucoma. Nevertheless, the importance of optic disc cupping should not be underrated. In terms of glaucoma screening, accurate risk assessment and stratification of eyes at risk for glaucoma are essential, and close monitoring of selected cases can improve the outcomes of glaucoma screening programs. The present study has a strength in that it demonstrated the long-term follow-up results of eyes with large optic disc cupping, i.e., so-called glaucoma-like discs (GLDs), and investigated the risk factors for conversion to glaucoma. In particular, in addition to CDRs, variable parameters, which have been demonstrated to be associated with glaucomatous damage and can be easily identified on screening fundus photography, were used to analyze individual risks for glaucoma conversion.

The present study demonstrated that eyes with vertical CDR $\ge 0.7$ were significantly associated with the risk of future glaucoma conversion. Other typical optic disc configuration changes, such as ISNT rule violation and vertical cupping, were also significant baseline risk factors for RNFLDs. These results imply that clinicians can find clues about ongoing glaucomatous damage before apparent RNFLDs are observed on fundus photography. In fact, nonhuman primate models of glaucoma have provided the insight that ONH surface height change precedes RNFL thinning^26,27^. The temporal relationship between ONH cupping and RNFL thinning was further confirmed by Xu et al., who demonstrated that ONH surface depression occurred before RNFL thinning by up to 41 months (with a median of 16 months)^28^. At the same time, large optic disc cupping has been well documented to be a significant risk factor for glaucoma in large, longitudinal epidemiologic studies. The OHTS study showed that baseline factors, including larger vertical or horizontal CDRs, old age, higher IOP, larger pattern standard deviations, and thinner central corneal measurements, predicted conversion to open-angle glaucoma (OAG)^13^. Another longitudinal population-based study from Ghana, the Tema Eye Survey, showed that a larger vertical CDR, male sex, older age, higher IOP, and thinner central corneal thickness (CCT) were significant baseline risk factors for incident OAG^14^. The present data are consistent with these previous findings, highlighting the importance of careful evaluation and monitoring of optic disc cupping in suspected cases.

The nasalization of CRVT ($\ge 60\%)$ was significantly associated with glaucoma conversion. Interestingly, a greater PPA area (PPA-to-DA ratio $\ge 0.4$) and tilted optic disc configuration (disc ovality $\ge 1.2)$ were also significant risk factors for RNFLDs. These findings are consistent with previous reports that investigated the effect of the location of CRVT on glaucoma susceptibility and PPA location. Jonas et al.^29^ reported that glaucomatous neuroretinal rim loss was dependent on the distance from the region of the affected rim to the CRVT. The location of the CRVT was also spatially correlated with the location of enlarged PPA: the longer that the distance to the CRVT exit was, the larger that the PPA was, and the smaller that the neuroretinal rim was^30^. Lee and colleagues recently proposed that the nasalization of CRVT is related to axial elongation in myopic eyes and demonstrated increased susceptibility to glaucomatous damage in these eyes^31^. During myopic axial elongation, the CRVT is dragged nasally, and the temporal border tissue of the ONH is further stretched temporally, resulting in an enlarged PPA and tilted optic disc configuration. The present longitudinal data support this idea, leading to incidental RNFLDs from increased ONH strain, as indicated by CRVT nasalization, larger PPA, and a tilted optic disc.

DH has been widely accepted to be associated with the development and progression of glaucoma^32,33^, although the development of DH is unpredictable. In line with this theory, DH was a significant, and most detrimental risk factor for incident RNFLDs and their progression in the present longitudinal observation. In fact, the predictive power of the aforementioned scoring system could be improved, since the best cutoff score was 7.1 with sensitivity of 61.1% and specificity of 90.4% (AUROC = 0.856), by including the presence of DH, which added 6.02 to the final score. This finding emphasizes that the detection of DH, along with at least one other risk sign, would be decisive evidence to anticipate glaucoma conversion in the future. Considering that DH can only be identified through careful examination of the ONH, clinicians should not underestimate the importance of monitoring with fundus photography.

Fundus photography has the advantage of being able to observe morphological changes in blood vessels. A number of large epidemiological studies have cross-sectionally evaluated retinal vascular changes and shown that decreased retinal vessel caliber was significantly associated with glaucoma^34–36^. It is controversial whether changes in the retinal vasculature are secondary findings due to decreased metabolic demands from RNFLDs or whether these retinal vasoconstrictions are primarily due to impaired local autoregulation and leakage of vasoactive substances^37^. The present study supports the role of vasospasm of retinal vessels in glaucoma development by showing that baseline changes in which blood vessels narrow or stiffen are significantly associated with the risk of future RNFLDs.

IOP elevation significantly increased the risk for glaucoma conversion. Eyes with baseline IOP $\ge 14$ mmHg had 1.70 times greater risk of incident RNFLD. Moreover, eyes with IOP fluctuation $\ge 2$ mmHg had a 1.50 times greater risk of progression with marginal significance (*P* = 0.08). In a 5-year, prospective, observational study involving normal-tension glaucoma eyes with IOP less than 15 mmHg, Sakata et al.^38^ revealed that long-term IOP fluctuation, along with DH and vertical CDR, was a significant risk factor for glaucoma progression, defined as VF deterioration or disc/peripapillary retina deterioration. The Advanced Glaucoma Intervention Study demonstrated a significant association between greater long-term IOP fluctuation and VF deterioration in the group with low mean IOP (10.8 mmHg) but not in the group with high mean IOP (20.6 mmHg)^39^. Given that the mean follow-up IOP in the current study population was not high (median value less than 15 mmHg), long-term IOP fluctuations could play an important role in glaucoma progression, especially in patients with a low range of IOPs.

The present study has the following shortcomings. First, the proposed baseline scoring system for predicting future glaucoma conversion must be further validated in a new study population to achieve more accurate diagnostic performance, although the current scoring system provides intuitive insight from baseline parameters to estimate the clinical outcomes of suspected cases. Second, unfortunately, we were not able to measure CCT during the period when the present study was conducted, although we currently obtain CCT value using a noncontact tonopachymeter which offers good feasibility for IOP as well as CCT measurement in health screening centers^40^. CCT has been well known as a risk factor for development of OAG, and therefore, might change the proposed scoring system. Third, the current study did not evaluate the VF of the subjects. Therefore, the present findings cannot be generalized to the prediction of functional outcomes of glaucoma because the progression of RNFLDs does not always mean the progression of VFs, especially in the very early stage of glaucoma. However, since structural progression usually precedes VF deterioration in glaucoma, the present findings still have strength in terms of the early detection of glaucomatous changes from suspected cases. Finally, with regard to assigning subjects to the control group, at least a 10-year duration of follow-up might not guarantee the absence of glaucoma conversion thereafter.

In conclusion, through long-term follow-up examinations, the present study demonstrated that some eyes with GLD (large optic disc cupping [vertical CDR ≥ 0.6] without RNFLDs) underwent conversion to glaucoma. Careful examination of screening fundus photography, including not only the ONH configuration but also retinal vascular changes, can predict the risk of glaucoma conversion and the progression of RNFLDs in these eyes. The current findings could provide clinicians with new insights to assess the risk of glaucoma in suspected cases and to identify patients who require close monitoring for glaucoma development. Although the proposed scoring system for predicting future glaucoma conversion should be further validated, we carefully suggest that subjects with high scores be followed up every 1–2 years while subjects with low scores be monitored every 3–5 years using screening fundus photography.

## Supplementary Information

Supplementary Information 1.

Supplementary Information 2.

Supplementary Information 3.

Supplementary Information 4.

Supplementary Information 5.

Supplementary Information 6.

Supplementary Information 7.

## Data Availability

The datasets generated during and/or analysed during the current study are available from the corresponding author on reasonable request.

## Abbreviations

AIC: Akaike information criterion
AUROC: Area under the receiver operating characteristic curve
CDR: Cup-to-disc ratio
CRVT: Central retinal vessel trunk
CSLO: Confocal scanning laser ophthalmoscopy
CWS: Cotton wool spot
DA: Disc area
DH: Disc hemorrhage
DM: Diabetes mellitus
HTN: Hypertension
ICC: Intraclass coefficient
IRB: Institutional Review Board
ISNT: Inferior > superior > nasal > temporal
LC: Lamina cribrosa
OAG: Open-angle glaucoma
OCT: Optical coherence tomography
OHTS: Ocular Hypertension Treatment Study
ONH: Optic nerve head
PACS: Picture archive and communication system
PPA: Peripapillary atrophy
RNFL: Retinal nerve fiber layer
RNFLD: Retinal nerve fiber layer defect
ROC: Receiver operating characteristic
SNUH: Seoul National University Hospital
VF: Visual field

## References

[1] Stein JD, Khawaja AP, Weizer JS (2021). Glaucoma in adults-screening, diagnosis, and management: A review. JAMA.

[2] Alencar LM, Zangwill LM, Weinreb RN (2010). A comparison of rates of change in neuroretinal rim area and retinal nerve fiber layer thickness in progressive glaucoma. Invest. Ophthalmol. Vis. Sci..

[3] Medeiros FA, Zangwill LM, Bowd C (2012). The structure and function relationship in glaucoma: Implications for detection of progression and measurement of rates of change. Invest. Ophthalmol. Vis. Sci..

[4] Abe RY, Diniz-Filho A, Zangwill LM (2016). The relative odds of progressing by structural and functional tests in glaucoma. Invest. Ophthalmol. Vis. Sci..

[5] Kass MA, Heuer DK, Higginbotham EJ (2002). The ocular hypertension treatment study: A randomized trial determines that topical ocular hypotensive medication delays or prevents the onset of primary open-angle glaucoma. Arch. Ophthalmol..

[6] Anderson DR (2003). Collaborative normal tension glaucoma study. Curr. Opin. Ophthalmol..

[7] Leske MC, Heijl A, Hussein M (2003). Factors for glaucoma progression and the effect of treatment: The early manifest glaucoma trial. Arch. Ophthalmol..

[8] Senthil S, Nakka M, Sachdeva V (2021). Glaucoma Mimickers: A major review of causes, diagnostic evaluation, and recommendations. Semin. Ophthalmol..

[9] Sihota R, Sidhu T, Dada T (2021). The role of clinical examination of the optic nerve head in glaucoma today. Curr. Opin. Ophthalmol..

[10] Quigley HA, Brown AE, Morrison JD, Drance SM (1990). The size and shape of the optic disc in normal human eyes. Arch. Ophthalmol..

[11] Jonas JB, Zäch FM, Gusek GC, Naumann GO (1989). Pseudoglaucomatous physiologic large cups. Am. J. Ophthalmol..

[12] Jonas JB, Budde WM, Panda-Jonas S (1999). Ophthalmoscopic evaluation of the optic nerve head. Surv. Ophthalmol..

[13] Gordon MO, Beiser JA, Brandt JD (2002). The ocular hypertension treatment study: baseline factors that predict the onset of primary open-angle glaucoma. Arch. Ophthalmol..

[14] Mwanza JC, Tulenko SE, Barton K (2019). Eight-year incidence of open-angle glaucoma in the tema eye survey. Ophthalmology.

[15] Hoyt WF, Frisén L, Newman NM (1973). Fundoscopy of nerve fiber layer defects in glaucoma. Invest. Ophthalmol..

[16] Jonas JB, Kling F, Gründler AE (1997). Optic disc shape, corneal astigmatism, and amblyopia. Ophthalmology.

[17] Tezel G, Trinkaus K, Wax MB (2004). Alterations in the morphology of lamina cribrosa pores in glaucomatous eyes. Br. J. Ophthalmol..

[18] Huang H, Jonas JB, Dai Y (2013). Position of the central retinal vessel trunk and pattern of remaining visual field in advanced glaucoma. Br. J. Ophthalmol..

[19] Bruce ASODJ, McKay D, Swann P (2007). Glaucoma — Vascular and nerve fibre layer signs. The Optician.

[20] Herschler J, Osher RH (1980). Baring of the circumlinear vessel. An early sign of optic nerve damage. Arch. Ophthalmol..

[21] Osher RH, Herschler J (1981). The significance of baring of the circumlinear vessel. A prospective study. Arch. Ophthalmol..

[22] Scheie HG (1953). Evaluation of ophthalmoscopic changes of hypertension and arteriolar sclerosis. AMA Arch. Ophthalmol..

[23] Hothorn T. maxstat: Maximally selected rank statistics. R package version 0.7–25. https://CRAN.R-project.org/package=maxstat. (2017).

[24] Robin X, Turck N, Hainard A (2011). pROC: An open-source package for R and S+ to analyze and compare ROC curves. BMC Bioinf..

[25] Youden WJ (1950). Index for rating diagnostic tests. Cancer.

[26] Strouthidis NG, Fortune B, Yang H (2011). Longitudinal change detected by spectral domain optical coherence tomography in the optic nerve head and peripapillary retina in experimental glaucoma. Invest. Ophthalmol. Vis. Sci..

[27] Fortune B, Burgoyne CF, Cull GA (2012). Structural and functional abnormalities of retinal ganglion cells measured in vivo at the onset of optic nerve head surface change in experimental glaucoma. Invest. Ophthalmol. Vis. Sci..

[28] Xu G, Weinreb RN, Leung CK (2014). Optic nerve head deformation in glaucoma: The temporal relationship between optic nerve head surface depression and retinal nerve fiber layer thinning. Ophthalmology.

[29] Jonas JB, Fernandez MC (1994). Shape of the neuroretinal rim and position of the central retinal vessels in glaucoma. Br. J. Ophthalmol..

[30] Jonas JB, Budde WM, Nemeth J (2001). Central retinal vessel trunk exit and location of glaucomatous parapapillary atrophy in glaucoma. Ophthalmology.

[31] Lee, K. M., Kim, M., Oh, S., Kim, S. H. Position of Central retinal vascular trunk and preferential location of glaucomatous damage in myopic normal-tension glaucoma. Ophthalmology Glaucoma (2018). 10.1016/j.ogla.2018.05.003 32672631

[32] Suh MH, Park KH (2014). Pathogenesis and clinical implications of optic disk hemorrhage in glaucoma. Surv. Ophthalmol..

[33] Kim KE, Park KH (2017). Optic disc hemorrhage in glaucoma: Pathophysiology and prognostic significance. Curr. Opin. Ophthalmol..

[34] Mitchell P, Leung H, Wang JJ (2005). Retinal vessel diameter and open-angle glaucoma: the Blue Mountains Eye Study. Ophthalmology.

[35] Amerasinghe N, Aung T, Cheung N (2008). Evidence of retinal vascular narrowing in glaucomatous eyes in an Asian population. Invest. Ophthalmol. Vis. Sci..

[36] Wang S, Xu L, Wang Y (2007). Retinal vessel diameter in normal and glaucomatous eyes: The Beijing eye study. Clin. Exp. Ophthalmol..

[37] Chan KKW, Tang F, Tham CCY (2017). Retinal vasculature in glaucoma: A review. BMJ Open Ophthalmol..

[38] Sakata R, Yoshitomi T, Iwase A (2019). Factors associated with progression of Japanese open-angle glaucoma with lower normal intraocular pressure. Ophthalmology.

[39] Caprioli J, Coleman AL (2008). Intraocular pressure fluctuation a risk factor for visual field progression at low intraocular pressures in the advanced glaucoma intervention study. Ophthalmology.

[40] Lee J, Choi HJ (2021). Accuracy and reliability of measurements obtained with a noncontact tono-pachymeter for clinical use in mass screening. Sci. Rep..

