# Supplementary material for: Screening fundus photography predicts and reveals risk factors for glaucoma conversion in eyes with large optic disc cupping
Source: Sci Rep. 2023 Jan 3;13:81. doi: 10.1038/s41598-022-26798-4 (PMC9810728; doi:10.1038/s41598-022-26798-4)
Supplement: Supplementary file 1 — Supplementary Information 1. [file 41598_2022_26798_MOESM1_ESM.docx]

**Supplementary Figure S1. Measurement of Optic Disc Configuration and Vascular Structure of the Optic Disc**

**(A)** The vertical and horizontal cup-to-disc ratio (CDR) were defined as the ratio of cup diameter to disc diameter on vertical (b_v_/a_v_) and horizontal meridians (b_h_/a_h_). Vertical cupping was defined when the vertical CDR was greater than the horizontal CDR. **(B)** Disc ovality is the ratio between the largest and smallest disc diameters (c/d). **(C)** Peripapillary atrophy area (PPA)-to-disc area (DA) ratio is the ratio of PPA area to DA (h/g). **(D)** The lamina cribrosa (LC) pore was determined to be visible if the eye allowed for clear visualization of the structural details of the LC surface (gray oval pores or fenestrae, black arrows). **(E)** Central retinal vessel trunk (CRTV) nasalization is estimated as the ratio of the distance between the CRVT and temporal disc border to the horizontal disc diameter (f/e). **(F)** The bayoneting of blood vessels was defined as the vessels being sharply bent or kinked while passing the edge of the cup (black arrows). **(G)** A circumlinear vessel (black arrows) lies superficially in the disc at the level of the retina and originates slightly above or below the central bifurcation of retinal vessels and curves to follow the contour of the physiologic cup, eventually to serve the macular regions. **(H)** The baring of circumlinear vessels (black arrows) was when the arteriole or vein fell to the bottom of the cup or molded onto the sidewall of the enlarging cup, rather than curving to follow the contour of the cup. **(I)** The fovea-disc angle (i) was defined as the angle between the horizontal line and the axis connecting the fovea to center of the optic disc on the acquired fundus image. It assumes a positive value when the fovea is located inferior to the optic disc center. **(J)** Vessel narrowing/sclerotic changes were determined by modifying Scheie’s classification system: diffuse arteriolar narrowing (arrow heads), focal constriction, arteriovenous compression (arrow), or copper-wire appearance were considered a positive sign.

**Supplementary Figure S2. ROC Curve of Baseline Scoring System for Prediction of RNFL Defect**

The areas under receiver operating characteristic (AUROC) of the risk score for the development of retinal nerve fiber layer (RNFL) defect was 0.856. The best cutoff score was 7.3, with sensitivity of 43.1% and specificity of 90.4%.
