# Supplementary material for: Screening fundus photography predicts and reveals risk factors for glaucoma conversion in eyes with large optic disc cupping
Source: Sci Rep. 2023 Jan 3;13:81. doi: 10.1038/s41598-022-26798-4 (PMC9810728; doi:10.1038/s41598-022-26798-4)
Supplement: Supplementary file 4 — Supplementary Information 4. [file 41598_2022_26798_MOESM4_ESM.docx]

**Supplementary Table S1. Interobserver Agreement for Parameters from Fundus Photography**

|  | **Cohen’s Kappa** | | **Agreement** |
| --- | --- | --- | --- |
| Vertical CDR $\geq$ 0.7 | 0.844 | | Almost perfect |
| Vertical cupping | 0.751 | | Substantial agreement |
| ISNT rule violation | 0.788 | | Substantial agreement |
| Bayonetting of blood vessels | 0.878 | | Almost perfect |
| Baring of circumlinear vessels | 0.813 | | Almost perfect |
|  | **ICC** | **95% CI** |  |
| Vertical CDR | 0.635 | 0.414 – 0.786 | Moderate |
| Disc ovality | 0.815 | 0.656 – 0.901 | Good |
| CRVT nasalization | 0.808 | 0.670 – 0.892 | Good |

CDR: cup-to-disc ratio, ISNT: inferior-superior-nasal-temporal, CRVT: central retinal vessel trunk, ICC: intraclass coefficient, CI: confidence interval
