# Supplementary material for: Screening fundus photography predicts and reveals risk factors for glaucoma conversion in eyes with large optic disc cupping
Source: Sci Rep. 2023 Jan 3;13:81. doi: 10.1038/s41598-022-26798-4 (PMC9810728; doi:10.1038/s41598-022-26798-4)
Supplement: Supplementary file 5 — Supplementary Information 5. [file 41598_2022_26798_MOESM5_ESM.docx]

**Supplementary Table S2. Cox Proportional Hazard Model for the Risk of RNFL Defect on the Fellow Eye**

| **Variable** | **HR (95% CI)** | ***P*-value** |
| --- | --- | --- |
| PPA to DA ratio$\geq$0.4 | 2.64 (0.99–7.01) | 0.05 |
| LC pore visibility | 2.08 (0.95–4.55) | 0.07 |
| **DH history** | **2.64 (1.19–5.86)** | **0.02** |
| Foveo-disc angle | 1.10 (0.99–1.22) | 0.06 |
| **Vessel narrowing/sclerotic change** | **4.64 (1.77–12.17)** | **0.002** |
| Mean IOP$\geq$13 mmHg | 2.42 (0.80–7.32) | 0.12 |

RNFL: retinal nerve fiber layer; HR: hazard ratio; CI: confidence interval; IOP: intraocular pressure; PPA: peripapillary atrophy; DA: disc area; LC: lamina cribrosa; DH: disc hemorrhage
