# Supplementary material for: Screening fundus photography predicts and reveals risk factors for glaucoma conversion in eyes with large optic disc cupping
Source: Sci Rep. 2023 Jan 3;13:81. doi: 10.1038/s41598-022-26798-4 (PMC9810728; doi:10.1038/s41598-022-26798-4)
Supplement: Supplementary file 6 — Supplementary Information 6. [file 41598_2022_26798_MOESM6_ESM.docx]

**Supplementary Table S3. Cox Proportional Hazard Model for the Risk of Progressive RNFL Thinning**

| **Variable** | **HR (95% CI)** | ***P*-value** |
| --- | --- | --- |
| **Bayoneting of blood vessels** | **0.51 (0.31–0.84)** | **0.009** |
| **DH history** | **2.32 (1.43–3.75)** | **<0.001** |
| **Cotton wool spot history** | **8.4E–09 (1.4E–09 – 4.9E–08)** | **<0.001** |
| IOP fluctuation$\geq$2 mmHg | 1.50 (0.95–2.39) | 0.08 |

RNFL: retinal nerve fiber layer; HR: hazard ratio; CI: confidence interval; FU: follow-up; IOP: intraocular pressure; Inf: infinity; DH: disc hemorrhage
