# Supplementary material for: Screening fundus photography predicts and reveals risk factors for glaucoma conversion in eyes with large optic disc cupping
Source: Sci Rep. 2023 Jan 3;13:81. doi: 10.1038/s41598-022-26798-4 (PMC9810728; doi:10.1038/s41598-022-26798-4)
Supplement: Supplementary file 7 — Supplementary Information 7. [file 41598_2022_26798_MOESM7_ESM.docx]

**Supplementary Table S4. Cox Proportional Hazard Model for the Risk of Glaucoma conversion with Baseline Factors**

| **Variable** | **HR (95% CI)** | ***P*-value** |
| --- | --- | --- |
| **Age** $\boldsymbol{\geq}$ **44 yrs** | **1.59 (1.01–2.49)** | **0.04** |
| Gender, female | 1.38 (0.89–2.12) | 0.15 |
| **Vertical CDR**$\boldsymbol{\geq}$**0.7** | **3.10 (1.81–5.31)** | **<0.001** |
| **Vertical cupping** | **1.62 (1.10–2.40)** | **0.01** |
| **ISNT rule violation** | **2.64 (1.25–5.17)** | **0.01** |
| **Disc ovality**$\boldsymbol{\geq}$**1.2** | **1.66 (1.07–2.56)** | **0.02** |
| **PPA to DA ratio**$\boldsymbol{\geq}$**0.4** | **1.63 (1.07–2.49)** | **0.02** |
| **CRVT nasalization**$\boldsymbol{\geq}$**60%** | **2.23 (1.44–3.48)** | **<0.001** |
| **Bayoneting of blood vessels** | **0.55 (0.36–0.84)** | **0.006** |
| **Vessel narrowing/sclerotic change** | **3.12 (1.98–4.92)** | **<0.001** |
| **Baseline IOP**$\boldsymbol{\geq}$**14 mmHg** | **1.60 (1.07–2.38)** | **0.02** |
| HTN | 0.74 (0.46–1.20) | 0.23 |

RNFL: retinal nerve fiber layer; HR: hazard ratio; FU: follow-up; IOP: intraocular pressure; CDR: cup-to-disc ratio; ISNT: inferior-supeior-nasal-temporal; PPA: peripapillary atrophy; DA: disc area; CRVT: central retinal vessel trunk
